# Granulin epithelin precursor promotes colorectal carcinogenesis by activating MARK/ERK pathway

**DOI:** 10.1186/s12967-018-1530-7

**Published:** 2018-06-04

**Authors:** Yi Pan, Siu Tim Cheung, Joanna Hung Man Tong, Ka Yee Tin, Wei Kang, Raymond Wai Ming Lung, Feng Wu, Hui Li, Simon Siu Man Ng, Tony Wing Chung Mak, Ka Fai To, Anthony Wing Hung Chan

**Affiliations:** 1 Department of Anatomical and Cellular Pathology, State Key Laboratory in Oncology in South China, Prince of Wales Hospital, The Chinese University of Hong Kong, 30-32 Ngan Shing Street, Shatin, NT, Hong Kong, SAR China; 2 Li Ka Shing Institute of Health Science, Sir Y.K. Pao Cancer Center, The Chinese University of Hong Kong, Hong Kong, SAR China; 3 Institute of Digestive Disease, Partner State Key Laboratory of Digestive Disease, The Chinese University of Hong Kong, Hong Kong, SAR China; 4 Division of Hepatobiliary and Pancreatic Surgery, Department of Surgery, Prince of Wales Hospital, The Chinese University of Hong Kong, Hong Kong, SAR China; 5 Division of Colorectal Surgery, Department of Surgery, Prince of Wales Hospital, The Chinese University of Hong Kong, Hong Kong, SAR China

**Keywords:** Colorectal cancer, GEP, Oncogene, MAPK/ERK pathway

## Abstract

**Background:**

Granulin epithelin precursor (GEP) is reported to function as a growth factor stimulating proliferation and migration, and conferring chemoresistance in many cancer types. However, the expression and functional roles of GEP in colorectal cancer (CRC) remain elusive. The aim of this study was thus to investigate the clinical significance of GEP in CRC and reveal the molecular mechanism of GEP in CRC initiation and progression.

**Methods:**

The mRNA expression of GEP in CRC cell lines were detected by qRT-PCR. The GEP protein expression was validated by immunohistochemistry in tissue microarray (TMA) including 190 CRC patient samples. The clinicopathological correlation analysis were achieved by GEP expression on TMA. Functional roles of GEP were determined by MTT proliferation, monolayer colony formation, cell invasion and migration and in vivo studies through siRNA/shRNA mediated knockdown assays. The cancer signaling pathway identification was acquired by flow cytometry, western blot and luciferase activity assays.

**Results:**

The mRNA expression of GEP in CRC was significantly higher than it in normal colon tissues. GEP protein was predominantly localized in the cytoplasm and most of the CRC cases demonstrated abundant GEP protein compared with non-tumorous tissues. GEP overexpression was associated with non-rectal location, advanced AJCC stage, regional lymph node and distant metastasis. By Kaplan–Meier survival analysis, GEP abundance served as a prognostic marker for worse survival in CRC patients. GEP knockdown exhibited anti-cancer effect such as inhibiting cell proliferation, monolayer colony formation, cell invasion and migration in DLD-1 and HCT 116 cells and decelerating xenograft formation in nude mice. siGEP also induced G1 cell cycle arrest and apoptosis. Luciferase activity assays further demonstrated GEP activation was involved in MAPK/ERK signaling pathway.

**Conclusion:**

In summary, we compressively delineate the oncogenic role of GEP in colorectal tumorigenesis by activating MAPK/ERK signaling pathway. GEP might serve as a useful prognostic biomarker and therapeutic target for CRC.

**Electronic supplementary material:**

The online version of this article (10.1186/s12967-018-1530-7) contains supplementary material, which is available to authorized users.

## Background

Colorectal cancer (CRC) is one of the major leading causes of cancer-related deaths in the world [1]. Although the overall survival of CRC patients has improved, the prognosis of patients with metastasis or recurrence is still relatively poor [2]. Angiogenesis is one of the major hallmarks of cancer [3], facilitating tumor development, progression and metastasis [4, 5]. Thus, understanding the molecular mechanism and identifying of novel molecular biomarker in angiogenesis could benefit clinical management of CRC patients.

Granulin epithelin precursor (GEP), also known as progranulin, acrogranin, proepithelin, and GP88/PC-cell derived growth factor, is a secreted glycoprotein composed of 7.5 repeats of cysteine-rich motif [6, 7]. Physiologically, it is expressed in immune cells [8, 9], neurons [10], epithelial cells [11] and chondrocytes [12], mediating wound healing, neurodegeneration and cartilage development [7, 10–12]. Pathologically, high expression levels of GEP are associated with poor prognosis in hepatocellular carcinoma, ovarian cancer [13], bladder cancer [14] and glioblastoma [15]. Functional studies reveals GEP acts as a growth factor to stimulate proliferation and migration, and confer chemoresistance in many types of cancers including breast cancer [16, 17], ovarian cancer [18, 19], liver cancer [20, 21] and bile duct cancer [22].

As the expression and functional role of GEP in CRC is unclear. In this study, the clinical significance and the function of GEP in CRC will be comprehensively revealed.

## Methods

### Patients and specimens

Between 1999 and 2013, 190 patients undergoing resection of primary CRCs at Prince of Wales Hospital, the Chinese University of Hong Kong were recruited in the present study. None of the patients received any neo-adjuvant therapy. The age of the patients ranged from 34 to 92 years, with a median age of 67.4 years. There were 106 men and 84 women. Tumors were staged according to the pathological tumor-node-metastasis (pTNM) staging system, 7th version. Distribution of the pTNM stages and other clinicopathological features is listed in Table 1. The patients were regularly followed up according to the institutional practice. Disease free survival was defined as the period from the date of curative surgery of primary tumor to the date that the patient survived without any signs or symptoms of that cancer. Overall survival was defined as the period from the date of curative surgery of primary tumor to the date of cancer-related death or last follow-up. The last update of the database 31 December 2015. The study was approved by Committees for Clinical Research Ethics of Joint Chinese University of Hong Kong-New Territories East Cluster.

**Table 1:** Clinicopathologic correlation of GEP expression in colorectal cancer (n = 190, significant *P*-value in italic format)

	GEP expression (n = 190)			*P*-value
	All	H-score ≥ 150	H-score < 150	
		98 (51.6%)	92 (48.4%)	
Male gender	106 (55.8%)	51 (52.0%)	55 (59.8%)	0.283
Age at operation (years, mean ± SD)	67.4 ± 12.1	66.7 ± 12.6	68.2 ± 11.6	0.386
Location				*0.032*
Right colon	51 (26.8%)	27 (29.0%)	24 (26.4%)	
Left colon	36 (18.9%)	23 (24.7%)	13 (14.3%)	
Rectum	88 (46.3%)	36 (38.7%)	52 (57.1%)	
Synchronous	9 (4.7%)	7 (7.5%)	2 (2.2%)	
Unknown	6 (3.2%)	/	/	
Size (cm, mean ± SD)	4.5 ± 1.7	4.6 ± 1.9	4.3 ± 1.6	0.257
Differentiation				
Well	2 (1.1%)	0	2 (2.2%)	0.052
Moderate	180 (94.7%)	92 (95.8%)	88 (97.8%)	
Poor	4 (2.1%)	4 (4.2%)	0	
Others	4 (2.1%)	/	/	
AJCC stage				< *0.01*
I	17 (8.9%)	5 (5.1%)	12 (13.0%)	
II	57 (30.0%)	19 (19.4%)	38 (41.3%)	
III	56 (29.5%)	27 (27.6%)	29 (31.5%)	
IV	60 (31.6%)	47 (48.0%)	13 (14.1%)	
T stage				0.583
T1	5 (2.6%)	3 (3.1%)	2 (2.2%)	
T2	19 (10.0%)	8 (8.2%)	11 (12.0%)	
T3	122 (64.2%)	61 (62.2%)	61 (66.3%)	
T4	44 (23.2%)	26 (26.5%)	18 (19.8%)	
N stage				< *0.01*
N0	89 (46.8%)	35 (35.7%)	54 (58.7%)	
N1	61 (32.1%)	34 (34.7%)	27 (29.3%)	
N2	40 (21.1%)	29 (29.6%)	11 (12.0%)	
M stage				< *0.01*
M0	129 (67.9%)	50 (51.0%)	79 (85.9%)	
M1	61 (32.1%)	48 (49.0%)	13 (14.1%)	
Pre-ops CEA (ng/ml, mean ± SD)	142.0 ± 838.4	218.9 ± 1118.1	53.4 ± 256.5	0.194

### Cell lines and cell culture

Human CRC cell lines Caco2, DLD-1, HCT 116, HT-29, LoVo, LS 180, SW480 and SW620 were all obtained American Type Culture Collection (ATCC, Manassas, VA, USA). All cell lines were cultured in media according to manufacturer’s instructions.

For transient transfection, DLD-1 or HCT 116 cells were transfected using lipofectamine 2000 (Thermo Fisher Scientific, Waltham, MA, USA) by siGEP or siRNA control (Qiagen, Hilden, Germany) to interfere GEP expression. Procedures of transfection were performed according to the manufacturer’s instructions. Cells stably expressing downregulated GEP mediated by shRNA was established using retrovirus system. The shRNA information for GEP knockdown can be found in Additional file 1.

### Quantitative reverse transcriptase-polymerase chain reaction (qRT-PCR)

Total RNA from cells was extracted by Trizol (Thermo Fisher Scientific) according to the manufacturer’s instruction. cDNAs were synthesized using High-Capacity cDNA Reverse Transcription Kit (Thermo Fisher Scientific) as protocol. Quantification was performed with the ABI 7500 Real Time PCR system (Applied Biosystems, Foster City, CA, USA). Primers and probes for GEP were GEP-forward (5′-CAA ATG GCC CAC AAC ACT GA-3′), GEP-reverse (5′-CCC TGA GAC GGT AAA GAT GCA-3′) and GEP-probe (5′-6FAM CCA CTG CTC TGC CGG CCA CTC MGBNFQ-3′). Primer and probe reagents for control 18s were ready-made reagents (Pre-Developed TaqMan Assay Reagents, Applied Biosystems). All experiments were performed in a minimum of three replicates.

### Immunohistochemistry (IHC)

Immunohistochemistry was performed on 5 µm sections cut from tissue microarray blocks using Dako Envision Plus System (Dako, Carpinteria, CA, USA) following the manufacturer’s instruction with modifications. Briefly, antigen retrieval was followed by endogenous peroxidase blocking and the following antibody in accordance with the manufacturer’s recommendations: GEP [23] (Clone: A23, 1:800). The cytoplasmic expression of was assessed by using histoscore (H-score) [24] for the staining intensity and the actual percentage of stained cells in the cytoplasm by two of the investigators. The H-score was obtained by the formula: 3 × percentage of strongly staining + 2 × percentage of moderately staining + percentage of weakly staining, giving a range of 0–300. The staining was considered positive when there was moderate or strong immunoreactivity over the cutoff point above 150.

### Western blot analysis

Equal total protein was separated by SDS-PAGE and transferred to nitrocellulose membrane. GEP was detected with a monoclonal anti-GEP antibody (1:5000 dilution). Other primary antibodies were from Cell Signaling (Danvers, MA, USA) commercially including Cyclin D1 (1:1000, #2926), cleaved PARP (Asp214) (1:1000, #9541), phospho-MAPK/ERK (1:2000, #9106), MAPK/ERK (1:1000, #9102), Caspase-8 (1:1000, #9746), Cleaved Caspase-8 (1:1000, #9748), Caspase-3 (1:1000, #9662), Cleaved Caspase-3 (1:1000, #9661). The secondary antibodies were anti-Mouse IgG-HRP (1:15,000, 00049039, Dako, Denmark) and anti-Rabbit IgG-HRP (1:5000, 00028856, Dako). The western blot bands were quantified by ImageJ.

### Cell proliferation assay

Cell proliferation assays were performed using 3-(4,5-dimethylthiazol-2-yl)-2,5-diphenylte-trazolium bromide (MTT, Sigma-Aldrich, St. Louis, MO, USA) assay following the manufactures’ protocol. Briefly, CRC cells which have been transfected with siGEP or siControl were plated in 96-well plate. Cell viability was measured at 24, 48, 72, 96, and 120 h. Finally, the optical density was determined at 570 and 690 nm wavelength light absorption (Victor3, Perken Elmer, MA, USA).

### Monolayer colony formation assay

Anchorage-dependent growth was assessed by monolayer colony formation. 6-well plates were used at a density of 1 × 10^3^ to 5 × 10^3^ for transfected cells. Cells were incubated at 37 °C for 14–21 days until colonies can obviously be observed. Colonies were fixed with methanol for 5 min and stained with 0.5% crystal violet. Colonies with cell numbers of more than 50 cells per colony were counted. The experiments were performed in duplicate wells in three independent experiments.

### Invasion and migration assay

Cell invasion or migration activity were performed using 24 well biocoat matrigel invasion chambers or sterilized transwell insert chambers (Corning, Bedford, MA, USA). Cells were harvested after transfected with siGEP or siControl for 24 h and re-plated with serum free medium in the upper chamber. The lower chamber was filled with culture medium containing 10% FBS as the chemoattractant. After 48 h, the cells that moved to the lower surface of polycarbonate membrane were stained with 0.5% crystal violet and counted at five random 200× fields.

### In vivo tumorigenic assays

1 × 10^6^ transfected CRC cells in 100 μl PBS were injected subcutaneously into the dorsal region of anaesthetized nude mice (5 mice/construct, control in left and treatment in right). When tumor was formed, tumor diameter was measured and documented every 3 days for 3 weeks. At the end of investigation, mice were sacrificed, and xenografts were collected for diameter check and weigh valuation. The animal handling and all experimental procedures were approved by the Department of Health of Hong Kong and the Animal Experimentation Ethics Committee, The Chinese University of Hong Kong.

### Flow cytometry analysis

Cells transfected with siGEP or siControl were harvested at 24, 48 or 72 h after transfection. Then live cells were incubated in the mixture of propidium iodide (PI) and annexin V-fluorescein isothiocyanate (Thermo Fisher Scientific) for apoptosis study. Harvested cells were fixed and incubated with PI and RNase A at 4 °C for cell cycle analysis. Finally, cells were determined by FACS Calibur Flow Cytometer (Becton, Dickinson and Company, Franklin Lakes, NJ, USA) and CellQuest program.

### Luciferase reporter assay

The firefly luciferase construct was co-transfected with Renilla luciferase vector (Qiagen) as a control into the siRNA treated cells. Dual Luciferase Reporter Assay System (Promega, Fitchburg, Wisconsin, USA) was employed to check the luciferase activity after 48 h’ transfection. The results were expressed as the ratio of firefly luciferase activity to renilla luciferase activity. Experiments were repeated in triplicate.

### Statistical analysis

The statistical analysis was performed using IBM SPSS Statistics (Version 19.0, Armonk, NY, USA). The expression level of GEP in paired non-tumor and tumor tissues was compared with paired Student’s t-test. Independent Student’s t-test was used to compare the mean value of any two groups. The Pearson χ^2^ test was used to analyze the association of target expression with clinicopathological parameters. Survival curves were drawn using the Kaplan–Meier method and compared by means of the log-rank test. Univariable and multivariable Cox proportional hazard regression models were used to analyze independent prognostic factors. *P* < 0.05 was considered statistically significant. *P* < 0.01 was considered highly statistically significant.

## Results

### GEP is overexpressed in CRC

**Fig. 1:**
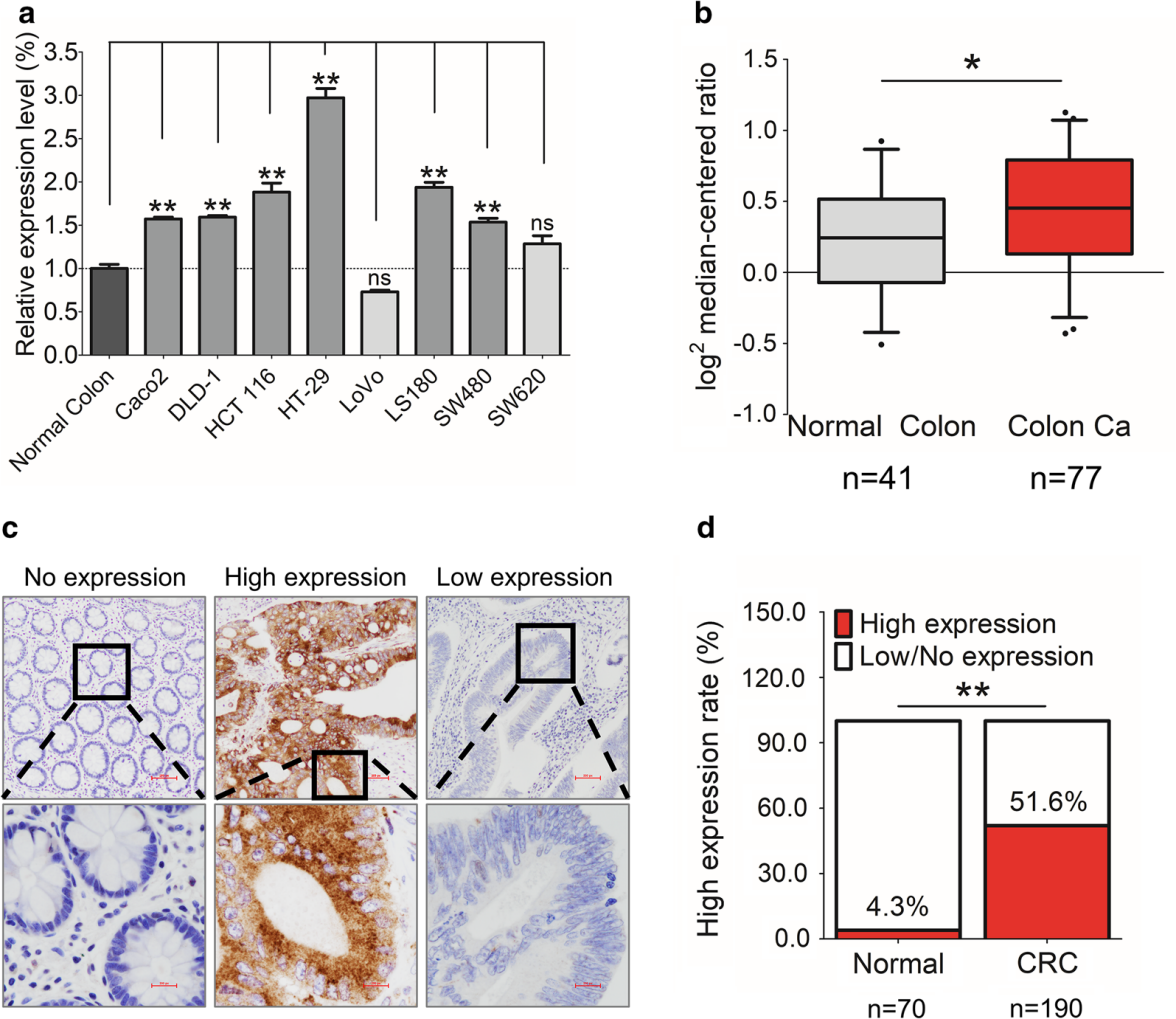
The expression of GEP in CRC. **a** GEP mRNA expression was significantly higher in CRC cell lines (6/8) than normal colon. **b** GEP mRNA expression was significantly higher in colon cancer tissues than the normal colon in the Ki colon dataset of ONCOMINE. **c** Representative IHC images showing no expression in normal colon (H-score = 0), high expression in tumor cells (H-score > 150), and low expression in tumor cells (H-score ≤ 150) of GEP in human CRC samples. **d** GEP protein expression was higher in primary CRCs (51.6%), compared to normal colonic mucosa (4.3%). (*P < 0.05; **P < 0.01)

GEP mRNA expression was up-regulated in 6/8 CRC cell lines compared with the normal colon epithelium by qRT-PCR (Fig. 1a). We analyzed GEP expression in cellular datasets available through ONCOMINE (http://www.oncomine.org/), an online collection of microarrays. Using the Ki colon dataset [7], we observed that GEP expression was lower in normal colon compared to colon cancer tissues (Fig. 1b). Using immunohistochemical staining on 190 CRC and 70 normal colon samples, GEP protein was predominantly localized in the cytoplasm and most of the CRC cases demonstrated abundant GEP protein compared with non-tumorous tissues (Fig. 1c). High expression (H-score ≥ 150) of GEP protein was detected in 51.6% (98/190) of tumor tissues. Whereas, only 4.3% (3/70) of normal tissues were found high GEP expression (*P *< 0.01, Fig. 1d).

### High GEP expression correlates with poor survival in CRC patients

**Fig. 2:**
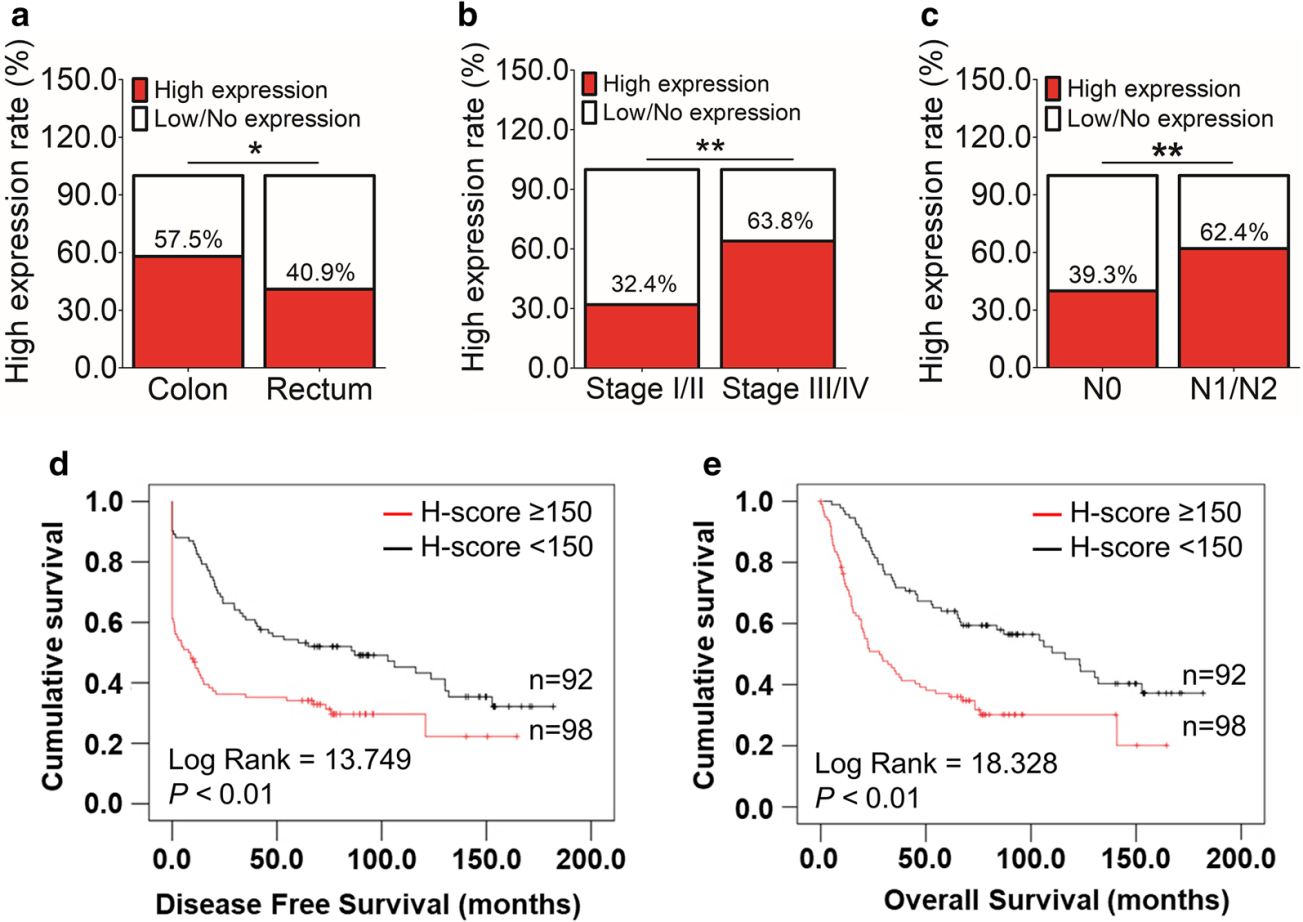
Clinicopathological and prognostic features of CRC patients with high GEP expression. **a** High GEP protein level was more frequently occurred in tumor located on colon than that on rectum (*P* < 0.05). **b** Higher GEP expression was also more commonly happened in patients with advanced AJCC stage (Stage III/IV, *P* < 0.01). **c** GEP upregulation was more involved in patients with lymphatic metastasis (*P* < 0.01). **d**, **e** GEP high expression correlated with both shorter disease-free survival (*P* < 0.01, **d**) and overall survival (*P* < 0.01, **e**). (*P < 0.05; **P < 0.01)

The correlation of GEP with clinicopathologic parameters in CRC patients was summarized in Table 1. GEP expression varied significantly among CRC samples with different tumor locations (*P* = 0.032), AJCC stages (*P* < 0.01), lymph node involvement (*P* < 0.01) and distant metastasis status (*P* < 0.01). Specifically, a high GEP protein level more frequently occurred in tumors located on colon than that on rectum (*P* < 0.05, Fig. 2a). High GEP expression was also more commonly happened in patients with advanced AJCC stage (Stage III/IV, *P* < 0.01, Fig. 2b), including the presence of lymphatic (*P* < 0.01, Fig. 2c) and distant metastasis (*P* < 0.01, Table 1).

High GEP expression was associated with shorter disease-free survival and overall survival (*P* < 0.01, Fig. 2d, e). Other Clinicopathological parameters such as right-side colon, poor differentiation, tumor invades adjacent organs or perforates the visceral peritoneum (T4), lymph node involvement (N1/2), distant metastasis (M1), and high CEA level before operation (> 10 ng/ml) also predicted worse disease-free survival and overall survival (Additional file 2). By multivariable Cox regression, compared with those classic prognostic parameters, high GEP expression was not an independent prognosticator for patients’ disease-free (Additional file 3) and overall survival (Additional file 4).

### GEP exerts an oncogenic function on CRC cells

**Fig. 3:**
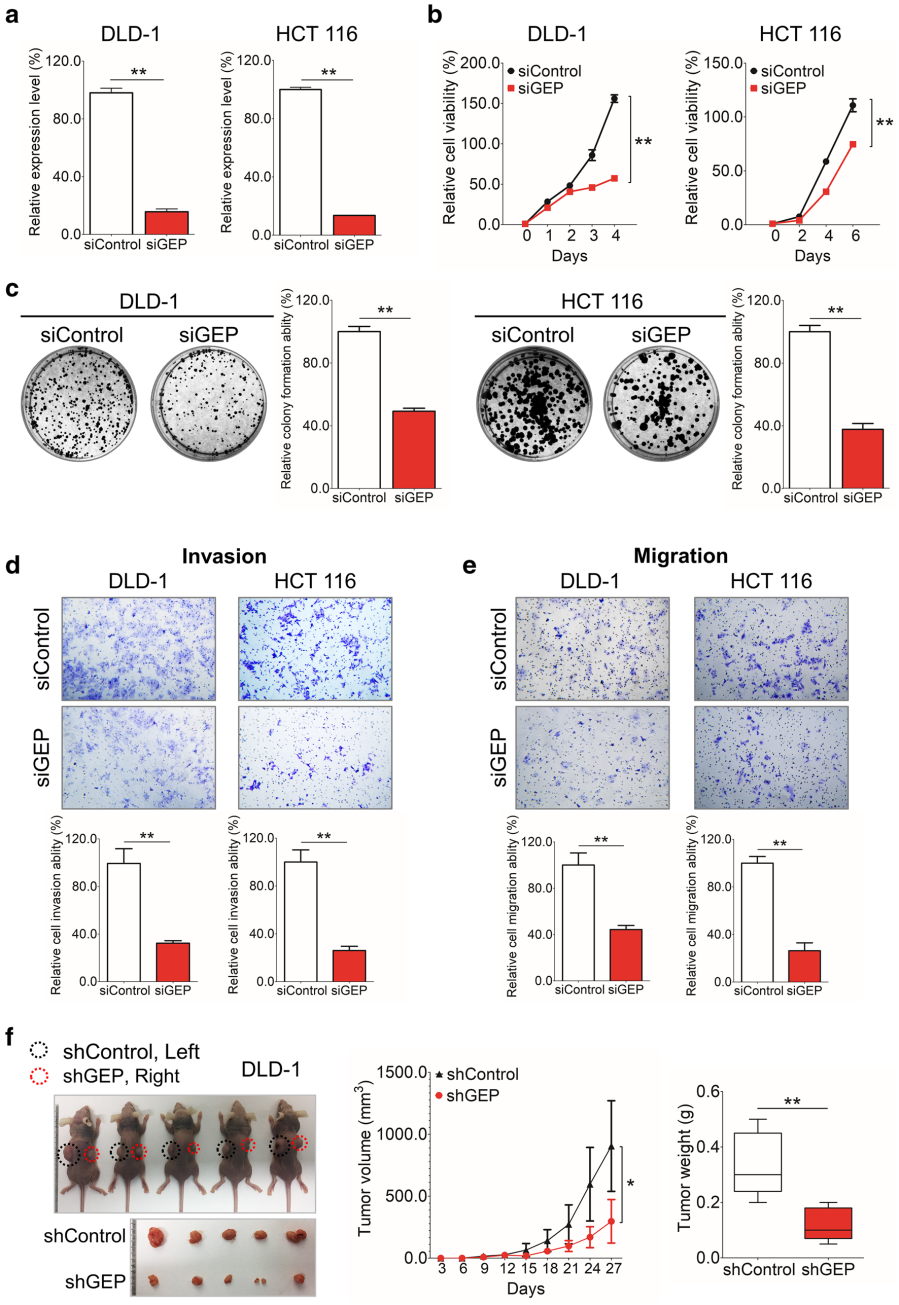
GEP exerts oncogenic function in CRC cells. **a** GEP showed decreased expression at the mRNA level by qRT-PCR in DLD-1 and HCT 116 cells. **b** A significantly decreased proliferation was observed in the siGEP treated group compared with siControl group in all 2 cell lines examined (*P* < 0.01). **c** GEP knockdown significantly reduced anchorage-dependent growth in CRC cell lines by Foci-Formation assay (*P* < 0.01). **d**, **e** Knockdown GEP expression by siRNA eliminated the ability of the and invasion (**d**) and migration (**e**) in both DLD-1 and HCT 116 cells compared to the control group. **f** Pictures of tumors isolated from nude mice at the end of investigation (Left); The tumor growth was monitored and calculated in the line chart (Middle) and histogram represented mean of the tumor weight (Right) from the shControl and shGEP groups. (*P < 0.05; **P < 0.01)

In DLD-1 and HCT 116 cell lines with endogenous high GEP expression, siRNA-mediated knockdown reduced GEP expression at mRNA (*P* < 0.01, Fig. 3a). siGEP treatments significantly decreased cell proliferation (Fig. 3b), anchorage-dependent growth (Fig. 3c), and abilities of the invasion (Fig. 3d) and migration (Fig. 3e) in both DLD-1 and HCT 116 cells compared to the control group. These observations suggested that GEP had oncogenic properties.

To determine the anti-tumor effect of shGEP in vivo, DLD-1 cells stably expressing GEP shRNA was established using retrovirus system. shControl and shGEP were subcutaneously injected into the left and right flanks of nude mice respectively. The shGEP group formed smaller tumor within 27 days (Fig. 3f). In contrast to shControl injected mice, the mice injected with shGEP exhibited greatly reduced mean tumor size (*P* < 0.05) and mean tumor weight (*P* < 0.01) in xenograft model (Fig. 3f).

### GEP knockdown results in G1 arrest and increased apoptosis in CRC

**Fig. 4:**
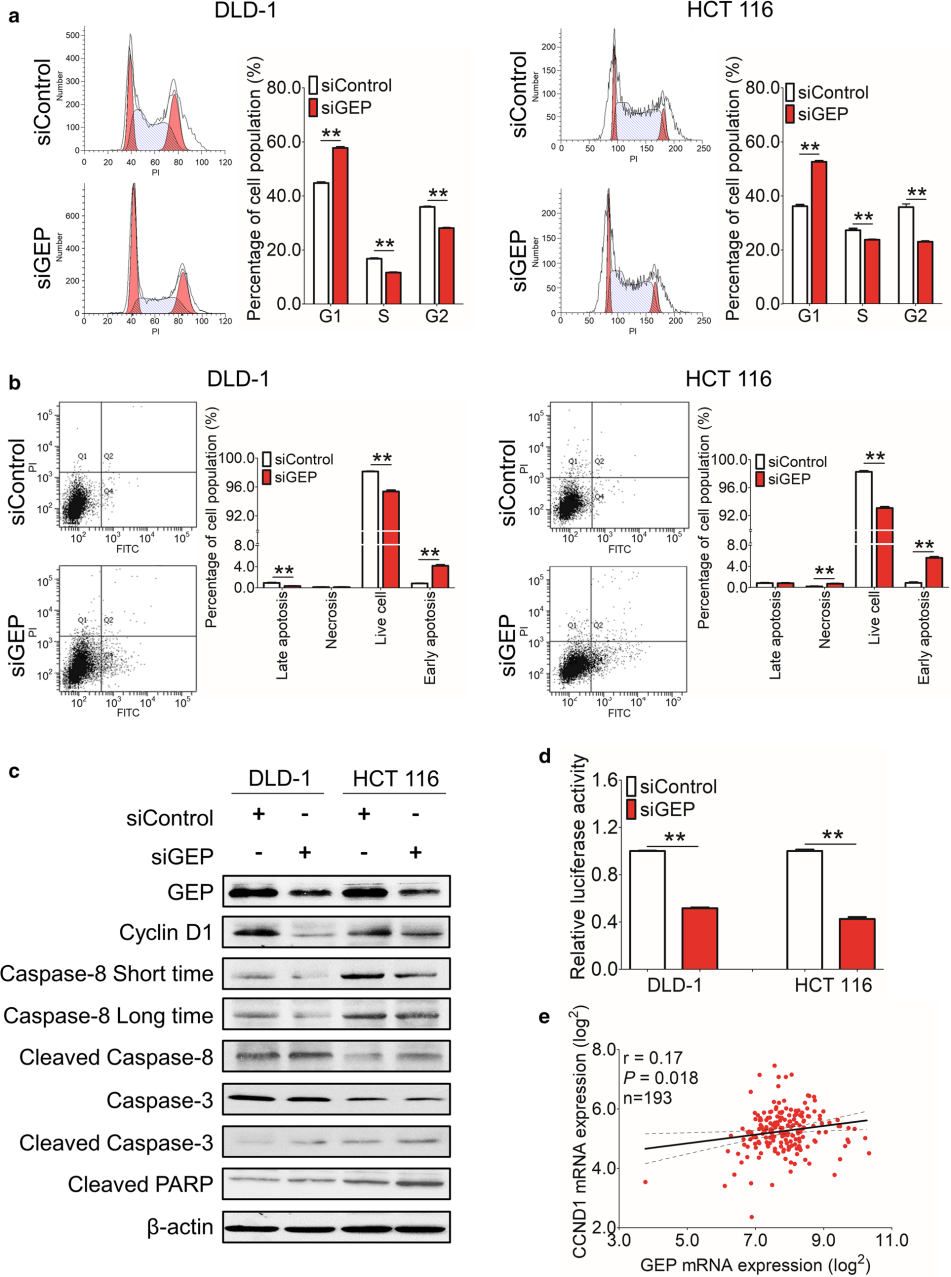
GEP knockdown results in G1 arrest and apoptosis in CRC. **a** Accumulation of G1 cells increased and S-phase cell percentage decreased in siGEP transfectants compared with siControls in DLD-1cell and HCT 116 cells lines. **b** The percentage of early apoptotic cells in siGEP treated cells was significantly increased compared to the siControl cells in these two cell lines. **c** Western blot of CyclinD1, activation of cleaved Caspase 3, activation of cleaved Caspase 8 and cleaved PARP expression after silencing GEP in DLD-1 and HCT 116 cells. **d** Relative luciferase reporter activity of cell cycle signaling shown in GEP suppressed DLD-1 and HCT 116 cells. **e** RNA expression of GEP was positively associated with CyclinD1 in TCGA database. (*P < 0.05; **P < 0.01)

Since a growth inhibitory effect was involved in siGEP transfected cells, we further explored the molecular basis involved in siGEP-suppressed tumor cell growth. We analyzed the transfectants for cell cycle parameters and apoptosis using flow cytometry. 24 h after transfection, accumulation of G1 cells increased in siGEP transfectant compared with siControls (57.8% vs. 44.8% in DLD-1; 52.6% vs. 36.2% in HCT 116 cells), while S-phase cell percentage decreased after siGEP transfection (11.6% vs. 16.8% in DLD-1; 23.8% vs. 27.3% in HCT 116 cells) in these 2 cell lines (Fig. 4a). For apoptosis analysis, the percentage of early apoptotic cells in siGEP treated cells was significantly increased compared to the siControl cells in both DLD-1 (0.8% ± 0.1% vs. 4.2% ± 0.2%, *P* < 0.01, Fig. 4b) and HCT 116 cells (0.8% ± 0.1% vs. 5.5% ± 0.2%, *P* < 0.01, Fig. 4b).

As proliferation-inhibition phenotypes were observed in siGEP groups, the associated cell cycle regulators and apoptosis markers were analyzed by western blot. Cyclin D1 was decreased in GEP knockdown cells, supporting the G0/G1-phase cell cycle arrest (Fig. 4c). Activation of cleaved Caspase 3, 8 and cleaved PARP were observed after silencing GEP expression (Fig. 4c), indicating that GEP inhibited apoptosis via a caspase-dependent pathway. Furthermore, luciferase reporter activity also showed GEP knockdown significantly suppressed cell cycle pathway in DLD-1and HCT 116 cells (Fig. 4d), confirming the flow cytometry and western blot result for cell cycle analysis. Besides, GEP expression was positively associated with CyclinD1 expression from RNA level in TCGA database (Fig. 4e).

### GEP promotes carcinogenesis via MAPK/ERK pathway in CRCs

**Fig. 5:**
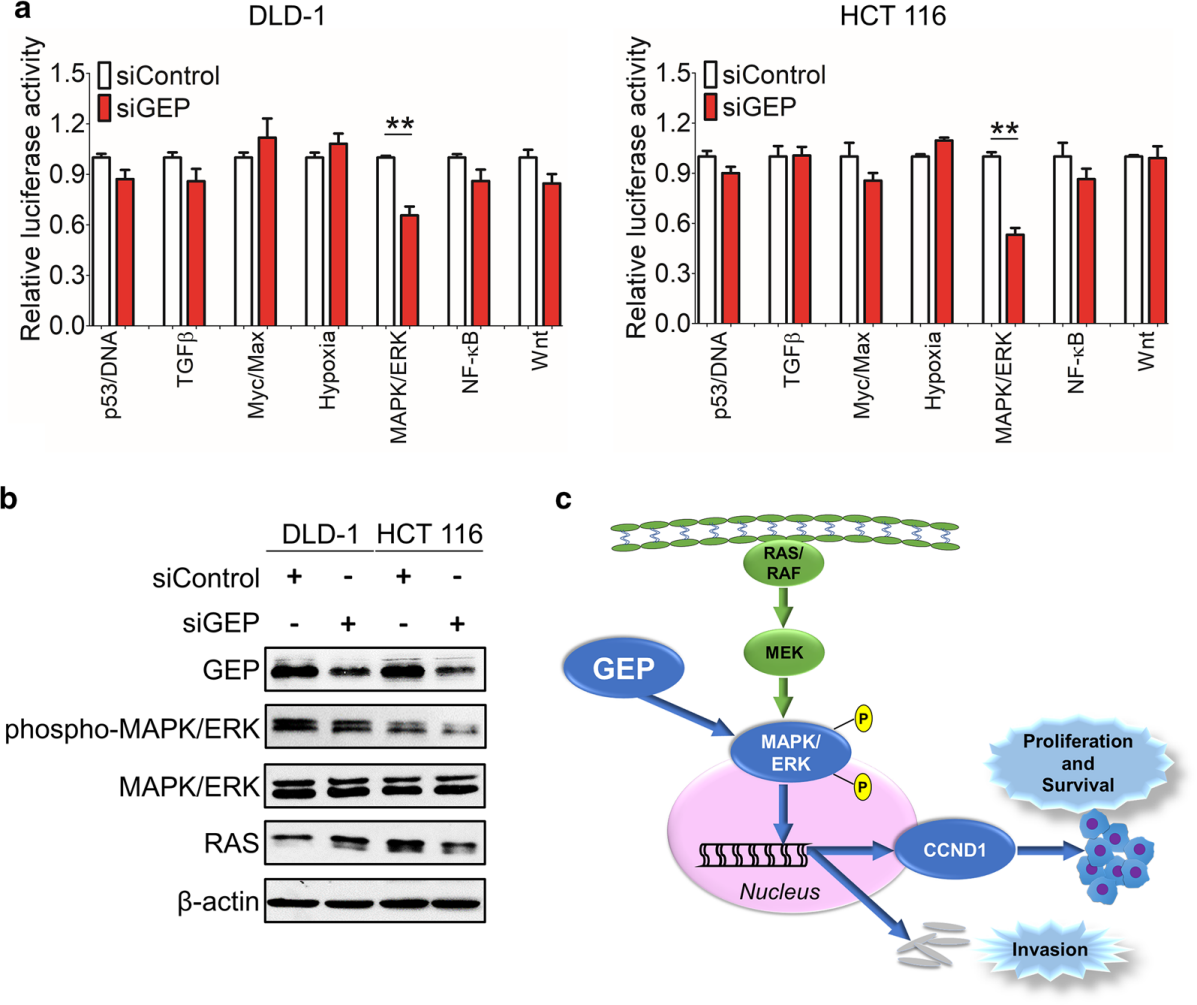
GEP promotes carcinogenesis via MAPK/ERK pathway in CRC. **a** A serial of promoter-luciferase assays (p53, TGFβ, Cell cycle, Myc, Hypoxia, MAPK/ERK, NF-κB and Wnt) were performed to screen for GEP target signaling pathways in DLD-1 and HCT 116 cells with GEP knockdown cells. **b** Western blot showed significant suppression of phospho-MAPK/ERK in the siGEP-treated CRC tumors cells compared with control. **c** GEP was shown to upregulate the phosphorylation of MAPK/ERK, nucleus translocation and stimulate cell proliferation, cell survival and metastasis of CRC. (*P < 0.05; **P < 0.01)

To further gain insights into the downstream signaling pathways modulated by GEP in CRC tumorigenesis, we examined the functional effect of GEP in several important cancer pathways including p53, TGFβ, Myc, Hypoxia, MAPK/ERK, NF-κB and Wnt by luciferase reporter activity assay. GEP knockdown significantly suppressed MAPK/ERK luciferase reporter activity in DLD-1 and HCT 116 cells (Fig. 5a). Western blot results showed significant suppression of phosphop-MAPK/ERK was observed in the siGEP treated CRC tumors (Fig. 5b), indicating that the MAPK/ERK signaling pathway was essential for anti-GEP-mediated growth inhibition in CRC cells. GEP led to MAPK/ERK phosphorylation and translocate into the nucleus, which stimulated cell proliferation, cell survival and metastasis of CRC (Fig. 5c).

## Discussion

In this study, we characterized clinicopathological features of GEP expression and delineated its oncogenic function in colorectal carcinogenesis.

GEP was overexpressed in CRC cell lines and patients’ tumor samples. Overexpression of GEP in CRC was associated with nodal and distant metastasis and poorer clinical outcome. These findings concurred with previous observations showing GEP overexpression in different human cancers including gliomas, renal, prostatic and hepatocellular carcinomas [25–28], and the correlation with poor survival [29]. Moreover, GEP expression was also positively associated with the MSI/CIMP subtypes of CRC in the TCGA cohort and patients with this kind of subtype had a very poor survival rate after relapse [30, 31] (Additional file 5). More importantly, increased DNA copy number of GEP could be detected in tumor samples in TCGA and Kurashina colon statistics from ONCOMINE (Additional file 6), indicating a strong possibility that overexpression of GEP was caused by copy number change from DNA level.

We further investigated the underlying mechanism of upregulating GEP on CRC. A series of in vitro and in vivo experiments confirmed GEP as an oncogenic factor in CRC. Notably, a decreased GEP level by RNA interference decreased the cell proliferation rate by MTT and colony-forming ability in the anchorage-dependent environment and decelerating xenograft formation in nude mice by disturbing cell cycle and apoptosis process and targeting the MAPK/ERK pathway. Thus, GEP played a crucial role in carcinogenesis of CRC. GEP expression has been reported in other aggressive tumors [26, 29] with a promoting role in cell growth regulation [32, 33], wound-healing process [34, 35], and murine development [36]. It has been previously shown that GEP binds with TNFR1/2 and diminish TNF-dependent activation of MAPK/ERK by altering the TNF/TNFR interaction [12]. Then it triggered activation of the MAPK/ERK and PI3K/AKT/PKB signaling cascades as well as focal adhesion kinase in the adhesion/motility pathway in cell lines derived from adrenal, breast, cervical, and bladder cancer cells, as well as in modified mouse embryo fibroblasts [16, 37–39]. Therefore, control of CRC growth by GEP could be also mediated through these pathways. A decreased level of MAPK/ERK phosphorylated form was observed in the GEP knockdown group from our study. Here, we also provided evidence that GEP promoted cell migration and invasion in CRC cells in vitro. A clinical association evaluation showed that overexpression of GEP was associated significantly with advanced AJCC stage, suggesting that overexpression of GEP in CRC may facilitate an invasive and metastatic phenotype.

GEP enhanced tumor cell survival, which was partly attributable to its anti-apoptotic ability. Defective apoptosis is one of the major causative factors in tumorigenesis and caspase activation has been considered a hallmark of apoptosis [40]. In this study, we demonstrated that knockdown of GEP induced cell apoptosis with caspase activation, suggesting that GEP modulated cell apoptosis via a caspase-dependent pathway. On the other hand, p53 was well-documented to play an important role in inducing cell apoptosis [41], and the inhibition of p53 may contribute to the anti-apoptotic effect of GEP. We found that GEP also exhibited the effect of apoptosis both in p53 wild type and mutated CRC cells, indicating that GEP promoted tumorigenesis regardless of p53 mutation status. In keeping with this, the p53 pathway was not significantly affected when GEP knockdown by luciferase reporter activity assay for p53 pathway analysis. Thus, GEP knockdown activated cell apoptosis in a p53 independent manner.

## Conclusions

Collectively, our findings demonstrated the oncogenic role of GEP in promoting tumorigenesis and metastasis in CRC. These findings suggest that GEP is a crucial factor in carcinogenesis of colon and rectum, and that GEP has the potential to serve as a prognostic marker and therapeutic target for CRC.

## Additional files

**Additional file 1.** SuperArray SureSilencing shRNA plasmids mediated GEP knockdown.

**Additional file 2.** Clinicopathological factors predicted prognosis for 190 CRC patients.

**Additional file 3.** Univariable and multivariable Cox regression of prognostic parameters for disease free survival in 190 patients with colorectal cancer.

**Additional file 4.** Univariable and multivariable Cox regression of prognostic parameters for overall survival in 190 patients with colorectal cancer.

**Additional file 5.** GEP mRNA expression was higher in MSI/CIMP subtype than other subtypes in TCGA cohort. (*P < 0.05).

**Additional file 6.** Copy number change of GEP in ONCOMINE colon tissue datasets. (*P < 0.05;* P* < 0.01).

## Abbreviations

CRC: colorectal cancer
GEP: granulin epithelin precursor
pTNM: pathological tumor-node-metastasis
ATCC: American Type Culture Collection
qRT-PCR: quantitative real-time polymerase chain reaction
TCGA: the cancer genome atlas
TMA: tissue microarray
IHC: immunohistochemistry
H-score: histoscore
MTT: 3-(4,5-dimethylthiazol-2-yl)-2,5-diphenylte-trazolium bromide
PI: propidium iodide

## References

[1] Torre LA, Bray F, Siegel RL, Ferlay J, Lortet-Tieulent J, Jemal A (2015). Global cancer statistics, 2012. CA Cancer J Clin.

[2] Punt CJA, Tol J (2009). OPINION More is less-combining targeted therapies in metastatic colorectal cancer. Nat Rev Clin Oncol.

[3] Hanahan D, Weinberg RA (2011). Hallmarks of cancer: the next generation. Cell.

[4] Ebos JM, Kerbel RS (2011). Antiangiogenic therapy: impact on invasion, disease progression, and metastasis. Nat Rev Clin Oncol.

[5] Dimova I, Popivanov G, Djonov V (2014). Angiogenesis in cancer—general pathways and their therapeutic implications. J BUON.

[6] Bateman A, Bennett HPJ (2009). The granulin gene family: from cancer to dementia. BioEssays.

[7] Liu CJ, Bosch X (2012). Progranulin: a growth factor, a novel TNFR ligand and a drug target. Pharmacol Ther.

[8] Jian J, Konopka J, Liu C (2013). Insights into the role of progranulin in immunity, infection, and inflammation. J Leukoc Biol.

[9] Arechavaleta-Velasco F, Perez-Juarez CE, Gerton GL, Diaz-Cueto L (2017). Progranulin and its biological effects in cancer. Med Oncol.

[10] Suzuki M, Nishiahara M (2002). Granulin precursor gene: a sex steroid-inducible gene involved in sexual differentiation of the rat brain. Mol Genet Metab.

[11] Zhu J, Nathan C, Jin WW, Sim D, Ashcroft GS, Wahl SM, Lacomis L, Erdjument-Bromage H, Tempst P, Wright CD, Ding AH (2002). Conversion of proepithelin to epithelins: roles of SLPI and elastase in host defense and wound repair. Cell.

[12] Tang W, Lu Y, Tian QY, Zhang Y, Guo FJ, Liu GY, Syed NM, Lai YJ, Lin EA, Kong L (2011). The growth factor progranulin binds to tnf receptors and is therapeutic against inflammatory arthritis in mice. Science.

[13] Han JJ, Yu MS, Houston N, Steinberg SM, Kohn EC (2011). Progranulin is a potential prognostic biomarker in advanced epithelial ovarian cancers (vol 120, pg 5, 2011). Gynecol Oncol.

[14] Selmy MA, Ibrahim GH, El Serafi TI, Ghobeish AA (2010). Evaluation of urinary proepithelin as a potential biomarker for bladder cancer detection and prognosis in Egyptian patients. Cancer Biomarkers.

[15] Wang MQ, Li G, Yin JY, Lin T, Zhang J (2012). Progranulin overexpression predicts overall survival in patients with glioblastoma. Med Oncol.

[16] Swamydas M, Nguyen D, Allen LD, Eddy J, Dreau D (2011). Progranulin stimulated by LPA promotes the migration of aggressive breast cancer cells. Cell Commun Adhes.

[17] Abrhale T, Brodie A, Sabnis G, Macedo L, Tian CS, Yue BB, Serrero G (2011). GP88 (PC-Cell Derived Growth Factor, progranulin) stimulates proliferation and confers letrozole resistance to aromatase overexpressing breast cancer cells. Bmc Cancer.

[18] Diaz-Cueto L, Arechavaleta-Velasco F, Diaz-Arizaga A, Dominguez-Lopez P, Robles-Flores M (2012). PKC signaling is involved in the regulation of progranulin (acrogranin/PC-Cell-derived growth factor/granulin–epithelin precursor) protein expression in human ovarian cancer cell lines. Int J Gynecol Cancer.

[19] Carlson AM, Maurer MJ, Goergen KM, Kalli KR, Erskine CL, Behrens MD, Knutson KL, Block MS (2013). Utility of progranulin and serum leukocyte protease inhibitor as diagnostic and prognostic biomarkers in ovarian cancer. Cancer Epidemiol Biomark Prev.

[20] Cheung ST, Cheung PFY, Cheng CKC, Wong NCL, Fan ST (2011). Granulin–epithelin precursor and ATP-dependent binding cassette (ABC) B5 regulate liver cancer cell chemoresistance. Gastroenterology.

[21] Cheung PFY, Cheng CKC, Wong NCL, Ho JCY, Yip CW, Lui VCH, Cheung ANY, Fan ST, Cheung ST (2011). Granulin–epithelin precursor is an oncofetal protein defining hepatic cancer stem cells. PLoS ONE.

[22] Frampton G, Invernizzi P, Bernuzzi F, Pae HY, Quinn M, Horvat D, Galindo C, Huang L, McMillin M, Cooper B (2012). Interleukin-6-driven progranulin expression increases cholangiocarcinoma growth by an Akt-dependent mechanism. Gut.

[23] Ho JC, Ip YC, Cheung ST, Lee YT, Chan KF, Wong SY, Fan ST (2008). Granulin–epithelin precursor as a therapeutic target for hepatocellular carcinoma. Hepatology.

[24] Choudhury KR, Yagle KJ, Swanson PE, Krohn KA, Rajendran JG (2010). A robust automated measure of average antibody staining in immunohistochemistry images. J Histochem Cytochem.

[25] Liau LM, Lallone RL, Seitz RS, Buznikov A, Gregg JP, Kornblum HI, Nelson SF, Bronstein JM (2000). Identification of a human glioma-associated growth factor gene, granulin, using differential immuno-absorption. Cancer Res.

[26] Donald CD, Laddu A, Chandham P, Lim SD, Cohen C, Amin M, Gerton GL, Marshall FF, Petros JA (2001). Expression of progranulin and the epithelin/granulin precursor acrogranin correlates with neoplastic state in renal epithelium. Anticancer Res.

[27] Pan CX, Kinch MS, Kiener PA, Langermann S, Serrero G, Sun L, Corvera J, Sweeney CJ, Li L, Zhang S (2004). PC cell-derived growth factor expression in prostatic intraepithelial neoplasia and prostatic adenocarcinoma. Clin Cancer Res.

[28] Cheung ST, Wong SY, Leung KL, Chen X, So S, Ng IO, Fan ST (2004). Granulin–epithelin precursor overexpression promotes growth and invasion of hepatocellular carcinoma. Clin Cancer Res.

[29] Jones MB, Michener CM, Blanchette JO, Kuznetsov VA, Raffeld M, Serrero G, Emmert-Buck MR, Petricoin EF, Krizman DB, Liotta LA, Kohn EC (2003). The granulin–epithelin precursor/PC-cell-derived growth factor is a growth factor for epithelial ovarian cancer. Clin Cancer Res.

[30] Gao JJ, Aksoy BA, Dogrusoz U, Dresdner G, Gross B, Sumer SO, Sun YC, Jacobsen A, Sinha R, Larsson E (2013). Integrative analysis of complex cancer genomics and clinical profiles using the cBioPortal. Sci Signaling.

[31] Cerami E, Gao JJ, Dogrusoz U, Gross BE, Sumer SO, Aksoy BA, Jacobsen A, Byrne CJ, Heuer ML, Larsson E (2012). The cBio cancer genomics portal: an open platform for exploring multidimensional cancer genomics data. Cancer Discov.

[32] Elkabets M, Gifford AM, Scheel C, Nilsson B, Reinhardt F, Bray MA, Carpenter AE, Jirström K, Magnusson K, Ebert BL (2011). Human tumors instigate granulin-expressing hematopoietic cells that promote malignancy by activating stromal fibroblasts in mice. J Clin Invest.

[33] Lu Y, Zheng L, Zhang W, Feng T, Liu J, Wang X, Yu Y, Qi M, Zhao W, Yu X, Tang W (2014). Growth factor progranulin contributes to cervical cancer cell proliferation and transformation in vivo and in vitro. Gynecol Oncol.

[34] He ZH, Ong CHP, Halper J, Bateman A (2003). Progranulin is a mediator of the wound response. Nat Med.

[35] Wang H, Sun Y, Liu S, Yu H, Li W, Zeng J, Chen C, Jia J (2011). Upregulation of progranulin by Helicobacter pylori in human gastric epithelial cells via p38MAPK and MEK1/2 signaling pathway: role in epithelial cell proliferation and migration. FEMS Immunol Med Microbiol.

[36] Kuse Y, Tsuruma K, Sugitani S, Izawa H, Ohno Y, Shimazawa M, Hara H (2016). Progranulin promotes the retinal precursor cell proliferation and the photoreceptor differentiation in the mouse retina. Sci Rep.

[37] Monami G, Gonzalez EM, Hellman M, Gomella LG, Baffa R, Iozzo RV, Morrione A (2006). Proepithelin promotes migration and invasion of 5637 bladder cancer cells through the activation of ERK1/2 and the formation of a paxillin/FAK/ERK complex. Can Res.

[38] Zhang XY, Pan ZX, Liu H, Yu JL, Li GX, Wang HY, Liu MM (2015). Effect of progranulin (PGRN) on the proliferation and senescence of cervical cancer cells. Genet Mol Res.

[39] Tanimoto R, Lu KG, Xu SQ, Buraschi S, Belfiore A, Iozzo RV, Morrione A (2016). Mechanisms of progranulin action and regulation in genitourinary cancers. Front Endocrinol (Lausanne).

[40] Johnstone RW, Ruefli AA, Lowe SW (2002). Apoptosis: a link between cancer genetics and chemotherapy. Cell.

[41] Galluzzi L, Morselli E, Kepp O, Tajeddine N, Kroemer G (2008). Targeting p53 to mitochondria for cancer therapy. Cell Cycle.

