# Supplementary material for: Granulin epithelin precursor promotes colorectal carcinogenesis by activating MARK/ERK pathway
Source: J Transl Med. 2018 Jun 4;16:150. doi: 10.1186/s12967-018-1530-7 (PMC5987413; doi:10.1186/s12967-018-1530-7)
Supplement: Supplementary file 1 — Additional file 1. SuperArray SureSilencing shRNA plasmids mediated GEP knockdown. [file 12967_2018_1530_MOESM1_ESM.docx]

**Additional file 1**

**SuperArray SureSilencing shRNA plasmids mediated GEP knockdown**

pGeneClipNeomycin Vector (~5kbp)

GEP shRNA

AGGCCCTGATAGTCAGTTCGAATTGACAGGAAGATTCGAACTGACTATCAGGGC

*Complement*

GCCCTGATAGTCAGTTCGAATCTTCCTGTCAATTCGAACTGACTATCAGGGCCT

shNC Scramble artificial sequence

AGGGAATCTCATTCGATGCATACTGACAGGAAGGTATGCATCGAATGAGATTCC

pGeneClips Neo sequence

CAGCTATGCATCCAACGCGTTGGGAGCTCCTCGAGGTTAACATCGATGCGGCCGCCCAGTCTACTTTTGAAACTCCAGACTGC
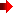
GAGATCTTGGGCCTCTGCCCCGACACAGCCTCATACGCCTCACTCTTTACACACACGGTCACTTGCCCCGCGCACTCCCGAGCCCTTTCCAGCCCTGACACACAGCTGGGATTCTCACTTCCGATCAGCGGTCCTGAACCCGCTCCCAGGGCACGGGAACTCCTTCGTGGTGAAGCAGCAAGTGGCGAAGCAGCAGCCTCTGCGCTGCCTCATCTACATAGAAGTCGCCCTGTCCGTGATGTCACCGACAGTGCCTTGCCCAGTCCCCGTCTGCCTTTCTGCCACTCAACCGACCAATCTGCTGCCAGAGCCGCCAAGGGGAAGTGACGTCTGCCTCTCCCTTTTTCCCTCCCGCCCCTGCGTCTGTTCTCTCCCAAAGAAGCTGGTCCTTANGGATCCGAATTCGTCGACTCTAGAGATATCGGGNCCAATTCGCCCTATAGTGAGTCGTATTACAATTCACTGGCCGTCGTTTTACACGTCGTGACTGGGAAAACCCTGGCGTTACCAACTTAATCGCCTTGCAGCACATCCCCCTTTCGCCAGCTGGCGTAATAGCGAANAGCCCGCACGATCGCCNTCCAACANTGCGCAGCTGAATGGGAATGGACCGCCCTGTACGGGCATTAGCGCGGNGGNGGGNGGTACCGCAAGNGACGCTAACTGCAGGCCCTANGCCGCTCTTTNCTTCTTCCTNCTTTCCGCANNNGNGGGTTTC

- insert position
