# Supplementary figures and images for: Granulin epithelin precursor promotes colorectal carcinogenesis by activating MARK/ERK pathway
Source: J Transl Med. 2018 Jun 4;16:150. doi: 10.1186/s12967-018-1530-7 (PMC5987413; doi:10.1186/s12967-018-1530-7)

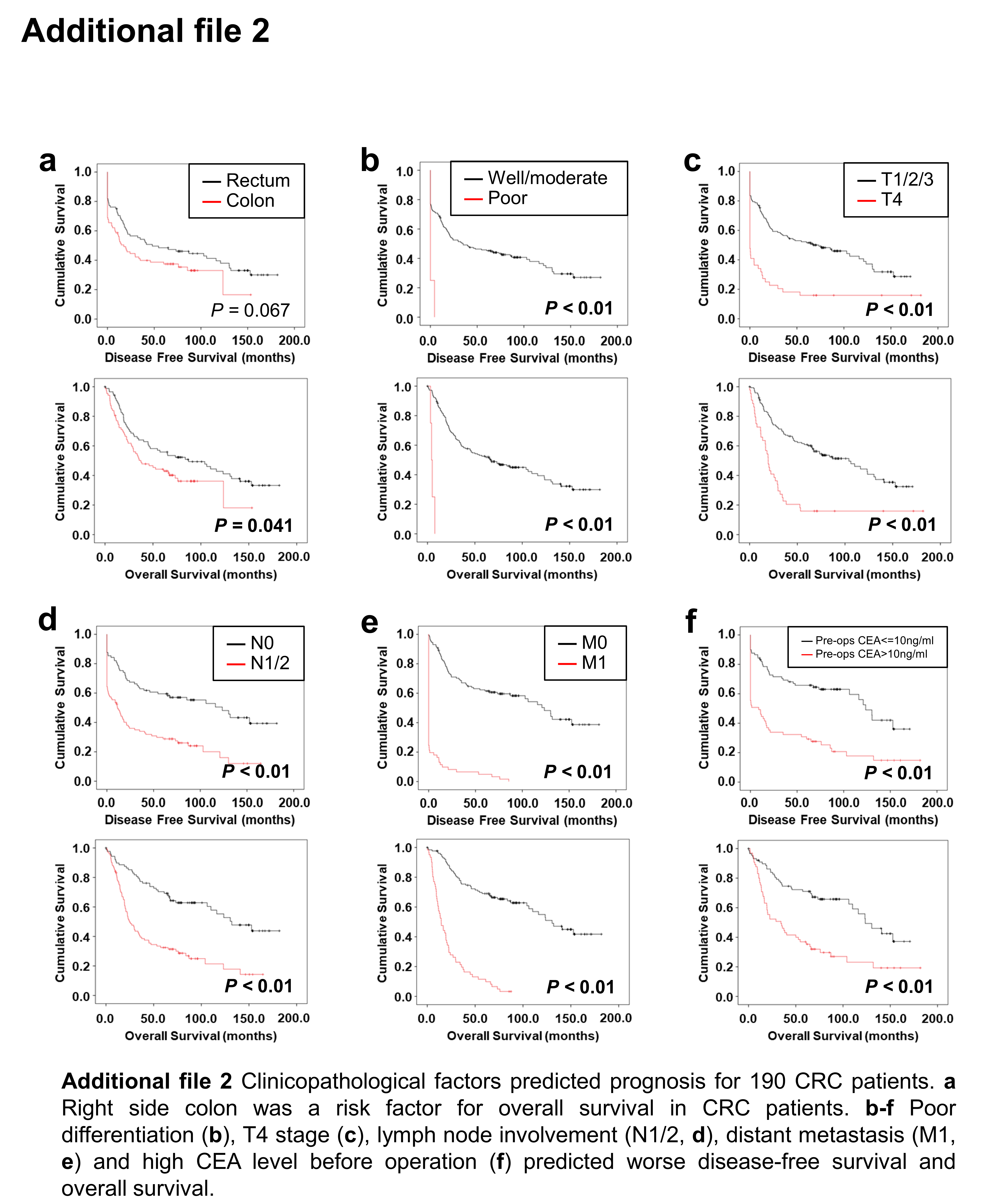

Supplement: Supplementary file 2 — Additional file 2. Clinicopathological factors predicted prognosis for 190 CRC patients. [file 12967_2018_1530_MOESM2_ESM.tif]

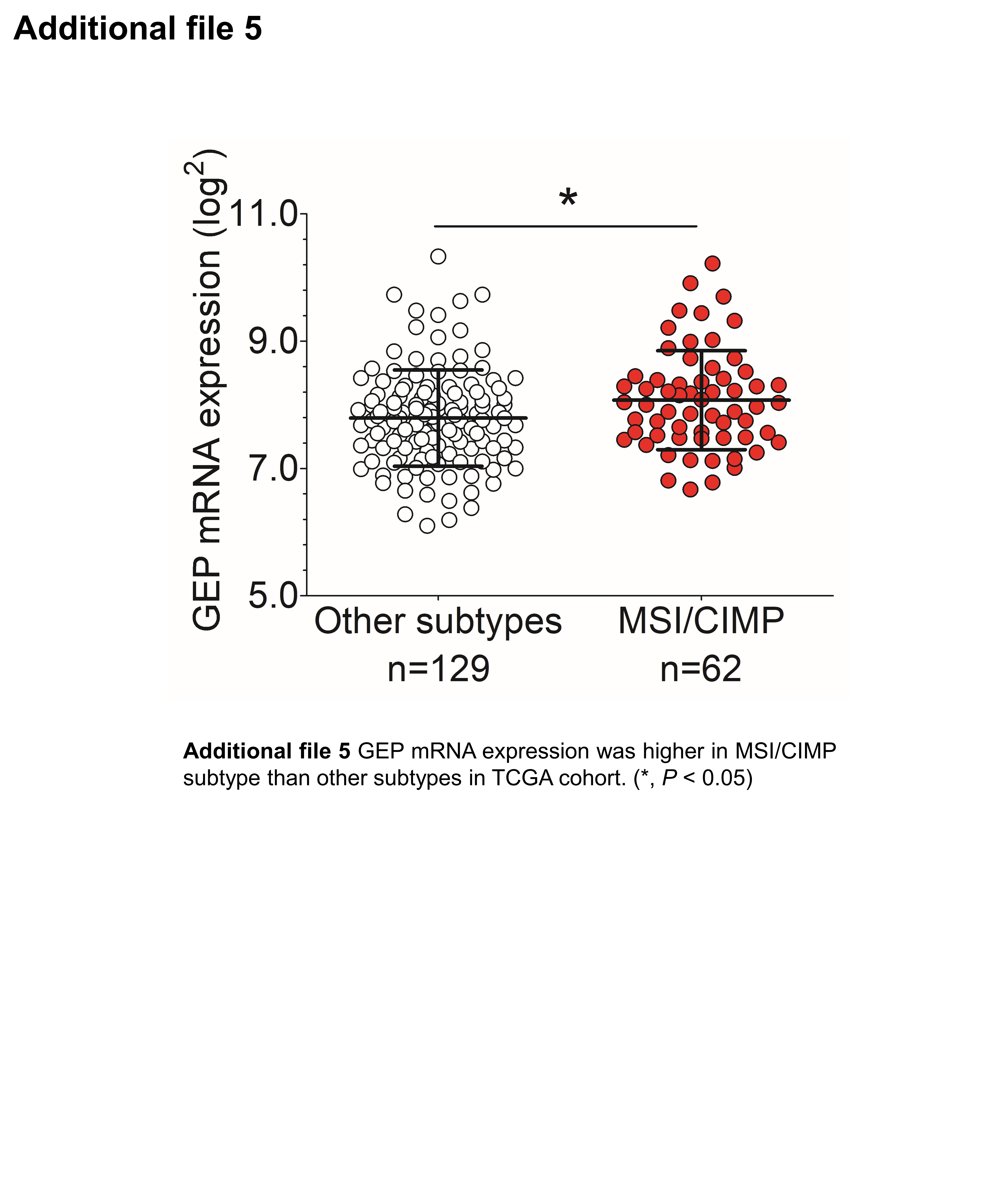

Supplement: Supplementary file 5 — Additional file 5. GEP mRNA expression was higher in MSI/CIMP subtype than other subtypes in TCGA cohort. (*P < 0.05). [file 12967_2018_1530_MOESM5_ESM.tif]

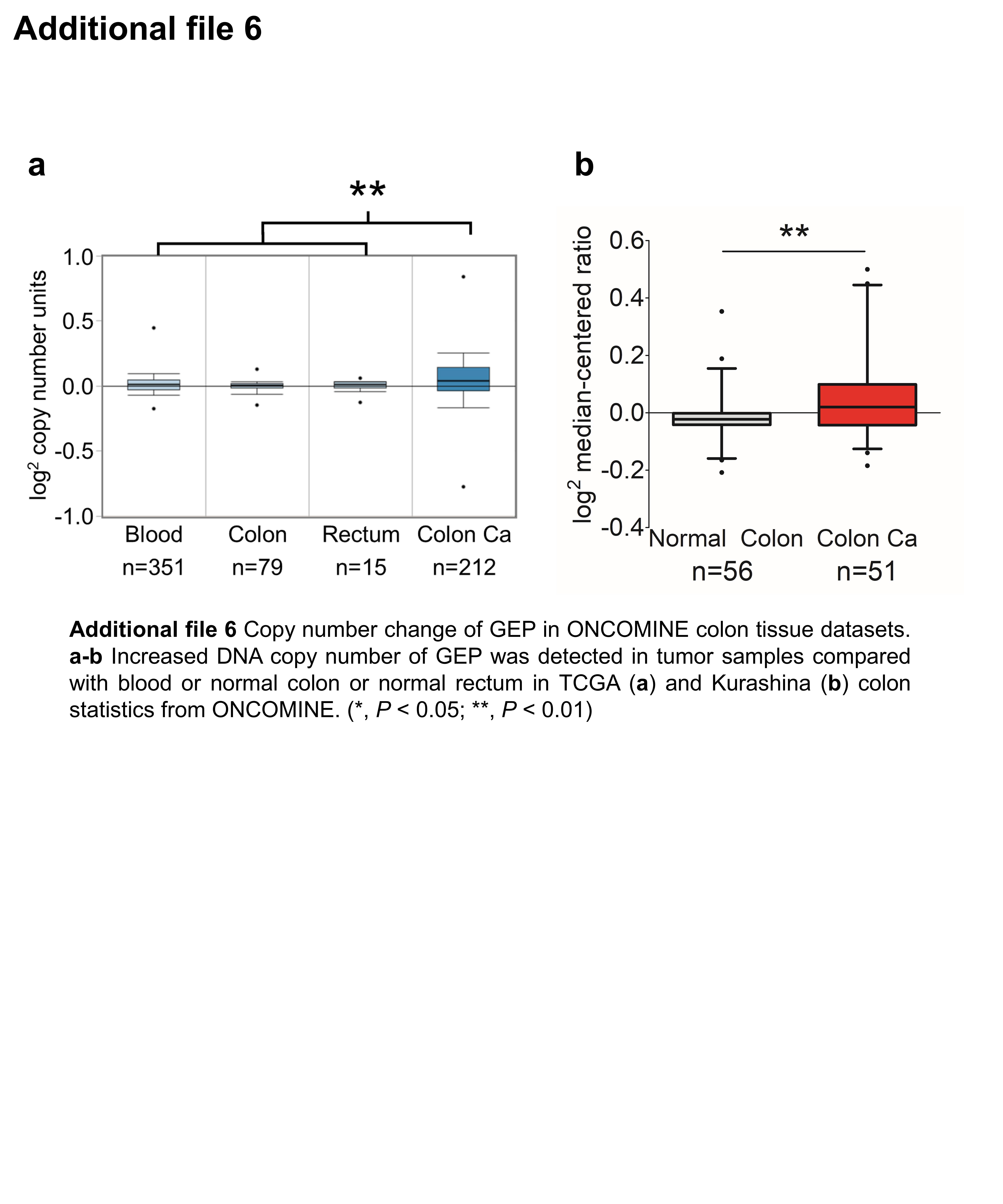

Supplement: Supplementary file 6 — Additional file 6. Copy number change of GEP in ONCOMINE colon tissue datasets. (*P < 0.05; P < 0.01). [file 12967_2018_1530_MOESM6_ESM.tif]
