# Supplementary material for: Granulin epithelin precursor promotes colorectal carcinogenesis by activating MARK/ERK pathway
Source: J Transl Med. 2018 Jun 4;16:150. doi: 10.1186/s12967-018-1530-7 (PMC5987413; doi:10.1186/s12967-018-1530-7)
Supplement: Supplementary file 3 — Additional file 3. Univariable and multivariable Cox regression of prognostic parameters for disease free survival in 190 patients with colorectal cancer. [file 12967_2018_1530_MOESM3_ESM.docx]

**Additional file 3**

**Additional file 3 Univariable and multivariable Cox regression of prognostic parameters for disease free survival in 190 patients with colorectal cancer.** (significant *P*-value in bold and Italic format).

| Disease free survival |  | Univariable |  |  |  | Multivariable |  |
| --- | --- | --- | --- | --- | --- | --- | --- |
|  | Hazard ratio | 95% CI | *P*-value |  | Hazard ratio | 95% CI | *P*-value |
| Male gender | 0.946 | 0.661-1.354 | 0.762 |  |  |  |  |
| Age at operation (years) | 1.000 | 0.985-1.015 | 0.979 |  |  |  |  |
| Location (colon *vs.* rectum) | 1.400 | 0.956-2.051 | 0.084 |  |  |  |  |
| Size (cm) | 1.062 | 0.963-1.172 | 0.231 |  |  |  |  |
| Differentiation (poor *vs.* well/moderate) | 4.085 | 1.472-11.338 | ***0.007*** |  | 1.310 | 0.396-4.325 | 0.658 |
| T stage (T3/T4 *vs.* T1/T2) | 1.727 | 0.948-3.146 | 0.074 |  |  |  |  |
| N stage (N1/N2 *vs.* N0) | 2.375 | 1.627-3.466 | ***<0.01*** |  | 1.631 | 1.022-2.602 | ***0.04*** |
| M stage (M1 *vs.* M0) | 8.605 | 5.667-13.066 | ***<0.01*** |  | 8.519 | 4.593-15.799 | ***<0.01*** |
| Pre-ops CEA >10 ng/ml | 2.672 | 1.754-4.069 | ***<0.01*** |  | 1.428 | 0.866-2.355 | 0.162 |
| GEP, H-score ≥150 | 1.916 | 1.326-2.770 | ***<0.01*** |  | 1.069 | 0.649-1.761 | 0.792 |
