# Supplementary material for: Granulin epithelin precursor promotes colorectal carcinogenesis by activating MARK/ERK pathway
Source: J Transl Med. 2018 Jun 4;16:150. doi: 10.1186/s12967-018-1530-7 (PMC5987413; doi:10.1186/s12967-018-1530-7)
Supplement: Supplementary file 4 — Additional file 4. Univariable and multivariable Cox regression of prognostic parameters for overall survival in 190 patients with colorectal cancer. [file 12967_2018_1530_MOESM4_ESM.docx]

**Additional file 4**

**Additional file 4 Univariable and multivariable Cox regression of prognostic parameters for overall survival in 190 patients with colorectal cancer.** (significant *P*-value in bold and Italic format).

| Overall survival |  | Univariable |  |  |  | Multivariable |  |
| --- | --- | --- | --- | --- | --- | --- | --- |
|  | Hazard ratio | 95% CI | *P*-value |  | Hazard ratio | 95% CI | *P*-value |
| Male gender | 0.988 | 0.682-1.433 | 0.951 |  |  |  |  |
| Age at operation (years) | 1.007 | 0.991-1.023 | 0.402 |  |  |  |  |
| Location (colon *vs.* rectum) | 1.509 | 1.015-2.245 | ***0.042*** |  | 0.890 | 0.524-1.512 | 0.667 |
| Size (cm) | 1.034 | 0.932-1.147 | 0.532 |  |  |  |  |
| Differentiation (poor *vs.* well/moderate) | 23.077 | 7.370-72.259 | ***<0.01*** |  | 5.470 | 1.450-20.635 | ***0.012*** |
| T stage (T3/T4 *vs.* T1/T2) | 1.722 | 0.922-3.217 | 0.088 |  | 1.504 | 0.730-3.101 | 0.269 |
| N stage (N1/N2 *vs.* N0) | 2.845 | 1.916-4.225 | ***<0.01*** |  | 1.547 | 0.912-2.626 | 0.106 |
| M stage (M1 *vs.* M0) | 7.053 | 4.698-10.588 | ***<0.01*** |  | 6.086 | 3.079-12.030 | ***<0.01*** |
| Pre-ops CEA >10 ng/ml | 2.562 | 1.662-3.950 | ***<0.01*** |  | 1.278 | 0.718-2.275 | 0.404 |
| GEP, H-score ≥150 | 2.256 | 1.540-3.304 | ***<0.01*** |  | 1.434 | 0.854-2.410 | 0.173 |
